# Human papillomavirus circulating tumor DNA assays as a mechanism for head and neck cancer equity in rural regions of the United States

**DOI:** 10.3389/fonc.2024.1373905

**Published:** 2024-05-08

**Authors:** Melina Windon, Catherine Haring

**Affiliations:** ^1^ Department of Otolaryngology-Head and Neck Surgery, University of Kentucky and Markey Cancer Center, Lexington, KY, United States; ^2^ Department of Otolaryngology-Head and Neck Surgery, The Ohio State University and the James Comprehensive Cancer Center, Columbus, OH, United States

**Keywords:** HPV, circulating tumor DNA, oropharyngeal cancer, rural cancer disparities, health equity, biomarker assay, liquid biomarker, cancer early detection

## Abstract

The rates of human papillomavirus-positive oropharyngeal cancer (HPV-OPC) are rising worldwide and in the United States, particularly in rural regions including Appalachia. Rural areas face unique health challenges resulting in higher cancer incidence and mortality rates, and this includes HPV-OPC. The recent advent of highly sensitive liquid biopsies for the non-invasive detection of HPV-OPC recurrence (circulating tumor HPV DNA, HPV ctDNA) has been swiftly adopted as part of surveillance paradigms. Though knowledge gaps persist regarding its use and clinical trials are ongoing, the ease of collection and cost-effectiveness of HPV ctDNA make it more accessible for HPV-OPC survivors than usual surveillance methods of frequent exams and imaging. Herein, we discuss how implementing HPV ctDNA assays in rural regions of the United States provide one poignant example of how liquid biopsies can improve cancer care equity.

## Human papillomavirus-associated oropharyngeal squamous cell carcinoma

Human papillomavirus (HPV) is the most common sexually transmitted infection in the United States and worldwide, and causes oropharyngeal, cervical, anal, penile, vulvar, and vaginal cancers. In recent years, oropharyngeal cancer (OPC) surpassed cervical cancer as the most common cancer type caused by HPV (1). There has been a dramatic increase in the incidence of OPC in the United States over recent decades, a trend that is attributable to HPV (2). Compared to head and neck cancers related primarily to tobacco use that are HPV-negative tumor status, HPV-positive oropharyngeal cancers (HPV-OPC) tend to occur among patients with less comorbidities, who are a few years younger (3, 4). HPV-positive tumor status is well established as a strong and independent positive prognostic factor in OPC (5), conferring a 5-year overall survival of around 80% compared to 40% (6). This survival difference, along with the improvements in treatment that have decreased treatment-related toxicity, has resulted in a growing population of survivors of this disease. Survivors of HPV-OPC represent a growing public health burden in the United States and worldwide (7). Importantly, there are higher rates of all HPV-associated cancers among rural versus urban populations, including HPV-OPC (8). Rural health communities face several unique health barriers that make them particularly susceptible to adverse oncologic outcomes. Herein, we demonstrate how liquid biopsy detecting high-risk HPV ctDNA as a biomarker of recurrence, persistence or metastasis represents a critical opportunity for improving health equity among patients with HPV-OPC.

## Cancer burden in rural regions of the United States

The United States Department of Health and Human Services has long recognized rural residence as a disparity contributing to poor health (9). Rural communities experience many challenges compared with non-rural areas, including lower rates of educational attainment, higher rates of poverty, and lack of access to healthcare services and public health infrastructure (10). These health challenges translate to higher cancer incidence and worse outcomes after cancer treatment. A recent analysis based on National Center for Health Statistics data compared cancer mortality in Kentucky and specifically Appalachian Kentucky compared to the rest of the United States. This group found that despite an overall improvement in mortality rates from year 1968 to 2018 in the United States, this trend was significantly attenuated in Kentucky and particularly in Appalachian Kentucky (11). Several other studies report higher cancer incidence and mortality rates in rural areas, including OPC (11–14).

The reasons for differential oncologic outcomes by rural residence are complex, but likely relate to differences in substance use, comorbidities, and most importantly, access to care. Rural communities have higher rates of tobacco and alcohol use, obesity, diet, poor oral health, and lack of physical activity and sun protective behaviors (13, 15). Reduced access to dental care represents an opportunity loss for oral cancer screening (15). Underdeveloped infrastructure and poor health facility access results in lower rates of screening and early detection for breast, colon, and lung cancer (16). Rural populations with cancer, including those with HPV-associated cancer, tend to present at a more advanced stage resulting in a decreased chance for cure (17).

There are also cancer treatment differences, with less access to tertiary academic centers and clinical trials (18). Longer distance from treating facilities impacts choice and adherence to recommended treatments (19). Financial barriers are a pressing concern in these regions, which have high rates of persistent poverty (20). Financial toxicity has been well described to have a significant impact on cancer survivorship, and is correlated with worse survival. Head and neck cancer patients experience among the highest rates of financial toxicity compared to patients with other cancer types. Among HPV-OPC specifically, this is thought to be due to the diagnosis among adults with relatively high function who experience opportunity losses related to unemployment or leave during their prime earning years (21, 22).

Time to initiation of adjuvant radiation therapy, a treatment included for most head and neck cancer patients with advanced disease, has long been recognized as a prognostic factor and a goal of initiation within six weeks following surgery is now considered a quality measure and guideline. Delays to initiation are more common among rural populations (23, 24).

Though HPV-OPC is a vaccine-preventable disease, the gap in both incidence and survival for HPV-OPC between rural and non-rural populations can be expected to widen not only because of the factors described above, but also because of low uptake of the HPV vaccine in these regions.

## Human papillomavirus vaccination

HPV vaccination is safe, recommended, and effective in reducing the incidence of both HPV-OPC (25) and cervical cancer (25, 26). However, projection modeling demonstrates that the HPV vaccine is not expected to curb the incidence of HPV-OPC among all comers until after at least the year 2045 (27), confirming the relevance of HPV-OPC as a public health issue at present. HPV vaccine uptake in the United States among adolescents is 63%, markedly lower than the Center for Disease Control’s goal of 80% (28). Rural areas have been shown to have a lower HPV vaccine uptake than urban communities (29). Purported reasons include lack of knowledge and distributed information about the vaccine, concern regarding its side effects absence of a healthcare personnel’s recommendation, cultural effects including concern the vaccine would increase promiscuity, fatalistic attitudes regarding the inevitability of cancer, safety concerns, high cost, and lack of parental and peer support (30–33).

The lower HPV vaccination rate reported in rural communities is concurrent with a broader national waning in HPV vaccine confidence (34, 35). This trend predates the COVID-19 pandemic, which had a further negative effect on public trust in preventative vaccination (36, 37). The rising HPV-OPC incidence and burgeoning vaccine hesitancy predicts that the geographically disproportionate burden of HPV-associated cancers should be expected to persist in rural regions. Considering the particular vulnerability of these regions to cancer-related health disturbance, a focus on improving the care of HPV-OPC patients in rural areas is acutely warranted.

## Surveillance after treatment for human papillomavirus-positive oropharyngeal cancer

Patients with HPV-OPC are currently treated with surgery or radiation alone, or combined, with the addition of chemotherapy for high-risk cancers. The majority of patients receive multimodality treatment. After treatment, patients are recommended to undergo rigorous surveillance. The National Comprehensive Cancer Network (NCCN) guidelines for head and neck cancer surveillance do not differ by HPV tumor status, despite HPV-OPC having improved prognosis, a longer disease-free interval, and distinct patterns of recurrence (38). Currently, the recommendation is frequent physical exams with an in-office endoscopy procedure every 1-3 months over the first year, 2-6 months in year two, and every 4-8 months during years 3-5. PET-CT is recommended following completion of systemic or radiation therapy, and anatomic cross-sectional imaging is recommended 3-4 months after surgery, with additional imaging when clinical concern for recurrence arises (39).

Notably, nearly one in four patients with HPV-OPC will recur (40). HPV tumor status remains a favorable prognostic factor in the recurrent/metastatic setting (41, 42), where treatment can improve survival and includes surgery, reirradiation with systemic therapy or immunotherapy as a single agent or in combination. Survival is better among those with distant metastatic disease when recurrence is oligometastatic (43) and amenable to surgical salvage (41, 44). Early detection of recurrence or metastasis when it is amenable to targeted therapy, therefore, is key to survival after recurrence.

Despite the recommended frequency of physical exams, several studies have demonstrated that most HPV-OPC recurrences are detected by imaging. This is distinct from HPV-negative OPC, where recurrence is more likely to be detected by patient symptoms or exam (38, 45). Imaging, however, still has limited sensitivity and specificity to detect recurrence.

The burden of surveillance is high on HPV-OPC survivors, particularly on rural patients. A recent survey study among a cohort of patients with HPV-OPC that drove a median of 57 miles to come to surveillance appointments found that almost two-thirds of patients surveyed were interested in a surveillance schedule that reduced the number of in-person clinic visits. Among this highly insured cohort, a significant burden of travel-related and missed opportunity costs was detailed (46). One can infer a greater burden caused by repeated in-person clinic visits in rural regions, where access to transportation, missed work and financial difficulties are prevalent barriers to medical care. Rural health status is characterized by delayed cancer detection, loss to follow up, and financial sensitivity to costly tests including PET-CT scans and flexible laryngoscopy.

There is ample room for improvement in the methods and schedule of HPV-OPC surveillance. With the advent of newly available liquid biopsies to detect recurrence of HPV-OPC, changes are anticipated to the current surveillance schedule to 1) improve detection of HPV-OPC recurrence and 2) reduce the burden of clinical exams and imaging, reflecting the relatively inferior sensitivity of these techniques.

## Biomarker testing in HPV-positive oropharyngeal cancer

**Figure 1:**
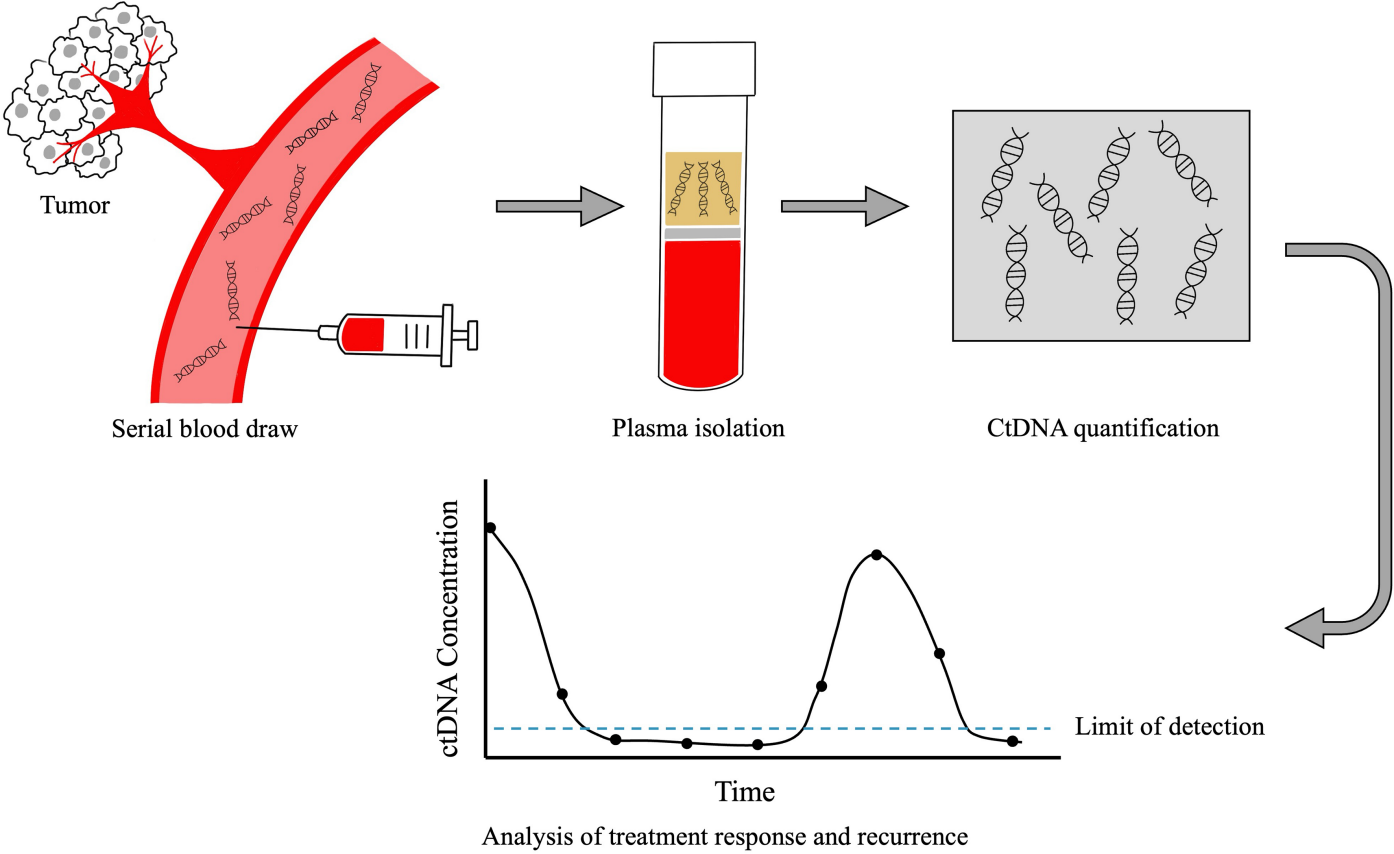
Technique for plasma CtDNA collection and analysis. After blood is collected from the patient, ctDNA is extracted from plasma. CtDNA is quantified and plotted over time. The absolute value of ctDNA correlates with disease burden, in that patients with high volumes of disease have higher ctDNA levels and those with lower disease burden have lower ctDNA levels. Changes in ctDNA levels over time reflect treatment response and recurrence prior to clinical detection by standard physical examination or imaging.

HPV-positive cancers are caused by high-risk oncogenic strains (types 16, 18, 31, 33, 35, 45, 53, etc) of the HPV DNA virus. The majority of oropharyngeal cancers are caused by HPV type 16. Given the prognostic implications associated with HPV status, it is considered standard of care to perform HPV testing on all tissue biopsies from patients with squamous cell carcinoma arising in the oropharynx (47). In recent years, it has been demonstrated that HPV circulating tumor (ct) DNA can also be detected in plasma in the majority of patients with HPV-OPC (48–50). As tumor cells progress through life cycle of replication, cell growth and apoptosis, tumor derived fragments of DNA enter the bloodstream, which can then be detected through various techniques including quantitative PCR, digital droplet PCR, and capture based next generation sequencing (**Figure 1**) (51–53). Currently, most commonly used HPV ctDNA assays are plasma-based, however data suggest that HPV ctDNA can also be detected in other bodily fluids including saliva (54) and urine (55). While test performance characteristics vary across studies, in general, HPV ctDNA testing has a sensitivity of 89-95% and specificity of 95-100% in detecting patients with untreated HPV-OPC (48, 50).

While HPV ctDNA tests have not yet undergone rigorous validation across institutions or in prospective clinical trials, a commercially available HPV ctDNA test now exists and is routinely used by providers given numerous studies demonstrating clinical validity (48, 53, 56). There is accumulating evidence suggesting that biochemical recurrence by ctDNA testing occurs months prior to clinical or radiographic recurrence (49, 56–58).

Most recurrences in OPC occur within two years of treatment completion, however patients with HPV-positive disease can recur at later disease-free intervals and have metastases to rarer organs (42), which is not detected with standard diagnostic algorithms. After definitive treatment, a PET-CT scan is obtained to assess treatment response during standard care. However, the positive predictive value of PET-CT scan in HPV-OPC is particularly low, frequently resulting in unnecessary additional work up or salvage surgery despite no residual disease (59, 60). A study by Tanaka et al. suggests that HPV ctDNA testing may outperform post-treatment PET-CT in determining clinical response, with superior positive and negative predictive values (61). These data suggest that incorporating HPV ctDNA testing into post-treatment decision-making may spare unnecessary procedures and salvage surgery in some patients.

In the post-treatment surveillance period, HPV ctDNA tests also have the potential to facilitate early detection of recurrence. Early detection of recurrence is associated with improved survival in OPC, as recurrences are more likely to be amenable to salvage surgery or radiation therapy (42, 44, 62). In a prospective study of 115 patients with HPV-OPC, 15 patients (13%) developed disease recurrence and HPV ctDNA testing was detected in most of these patients prior to recurrence diagnosis with a lead time ranging from 0-13 months. Additionally, in all patients with undetectable HPV ctDNA during surveillance, none developed recurrence (negative predictive value= 100%) (56). Importantly, HPV ctDNA testing may allow for early detection of distant metastases, which otherwise would not be detected with routine surveillance (57).

Many clinical trials are being designed to assess the validity of liquid biopsy assays with or without a concurrent reduction in surveillance schedules. HPV-OPC is one disease entity which exemplifies where a less burdensome means of surveillance can serve as a tool to improve health equity.

## Circulating tumor HPV DNA testing to promote health equity

HPV ctDNA testing can promote heath equity in HPV-OPC in three key ways: cost effectiveness, improved access to care, and early detection of recurrence.

Cost effectiveness analyses have been published demonstrating that the incorporation of ctDNA into surveillance schedules is economically favorable, when used to replace repeated post-treatment imaging (63, 64). The cost of the assay itself is far lower than tissue sampling and cross-sectional imaging, and so can be repeated in equivocal cases and easily trended over time to reduce the need for serial imaging.

Related to access, blood or urine samples are easier to procure than a clinic visit or imaging. The former requires either home collection in the case of urine (55), or a visit to a laboratory for a blood draw. A clinic visit requires time off work at the predetermined available appointment times that are offered, and usually longer travel. Imaging requires travel to the imaging center, and PET-CT scans require activity and diet restrictions.

Lastly, ctDNA testing may allow for early detection of recurrence. Studies reliably demonstrate that biochemical recurrence occurs months prior to clinical or radiographic recurrence. This diagnostic lead time theoretically allows an additional opportunity for engagement of the provider and patient, reducing the risk of loss to follow up and delays with scheduling and obtaining scans. Early detection of recurrence may allow for targeted therapy when the disease is localized, ultimately improving survival.

Though there are no current recommendations for population screening for HPV-OPC, further refinement of high-risk groups (65) that may benefit from screening could potentially allow for the use of ctDNA for screening in the future. For example, a recent study applied ctDNA testing to patients with clinical findings concerning for OPC, and correctly identified HPV-OPC in a subset of patients with positive testing (66). This strategy could expedite cancer diagnosis and workup, a key advantage in patients that face transportation barriers to returning to the clinic or hospital.

## Discussion

Though the data supporting HPV ctDNA as an actionable biomarker is very promising, there are additional steps that need to be taken to incorporate its use. It remains unclear how to optimally work up a survivor with detectable HPV ctDNA in order to localize and pathologically confirm a recurrence. And, according to the largest cohort study published to date, the sensitivity of HPV ctDNA for recurrence is 92.5% when considered on a per-test basis, and 87.3% when considered on a per-patient basis (67, 68). These results are very favorable, but HPV-OPC can indeed recur without a corresponding or preceding spike in ctDNA. Furthermore, not all HPV-OPC patients have detectable pretreatment ctDNA, diminishing its utility as a meaningful biomarker in surveillance in these patients. In patients with false negative ctDNA results at diagnosis or suspected recurrence, conventional surveillance methods of routine exam and imaging will continue to be relied upon. Next-generation sequencing, an as-yet emerging analytic platform, is more sensitive than the commercially available assays utilizing digital droplet PCR, and may be a useful assay in patients with otherwise undetectable pretreatment levels of HPV ctDNA (69).

The adoption of ctDNA into survivorship protocols needs to be supported with data. Ideally, HPV ctDNA assays and the subsequent diagnostic workup will be studied along with standard of care in a prospective setting order to ensure that overall survival is at least maintained. In rural populations, it is tempting to defer in-patient exams and imaging given the challenges this population faces. Though better than loss to follow up, HPV ctDNA assays are still inferior to a comprehensive surveillance plan that uses a thoughtful combination of lab testing, exams, imaging and liquid biopsy to augment and contextualize concerning findings.

In conclusion, rural populations in the United States face both a disproportionately high rate of HPV-OPC and several barriers to accessing cancer care. The development of HPV ctDNA assays for diagnosis, treatment response and surveillance for recurrence is a landmark advancement in HPV-OPC. The ability to improve access by using a blood or urine based assay to assist with diagnosis and augment and potentially deintensify surveillance schedules holds ample potential for improving cancer-related health equity, particularly among rural populations.

## Author contributions

MW: Writing – original draft, Writing – review & editing. CH: Writing – original draft, Writing – review & editing.
